# Evidence for the Prognostic Value of CDH17 Expression in Colorectal Carcinoma

**DOI:** 10.3390/ijms26146960

**Published:** 2025-07-20

**Authors:** Victor Ianole, Simona-Eliza Giușcă, Irina-Draga Căruntu

**Affiliations:** 1 Department of Morpho-Functional Sciences I, “Grigore T. Popa” University of Medicine and Pharmacy Iași, 16 University Street, 700115 Iasi, Romania; ianole.victor@umfiasi.ro (V.I.); irinadragacaruntu@gmail.com (I.-D.C.); 2 Pathology Department, “Prof. Dr. Nicolae Oblu” Emergency Clinical Hospital, 2 Ateneului Street, 700309 Iasi, Romania; 3 Pathology Department, “Dr. C. I. Parhon” Clinical Hospital, 50 Carol I Boulevard, 700503 Iasi, Romania; 4 Romanian Medical Science Academy, 1 I.C. Bratianu Boulevard, 030171 Bucharest, Romania

**Keywords:** CDH17, liver-intestine cadherin, colorectal cancer, immunohistochemistry, tumor emboli, lymphovascular invasion, prognosis, survival, biomarker, adhesion molecules

## Abstract

Colorectal cancer (CRC) diagnosed in an advanced stage has an increased predisposition for invasion and metastasis, requiring upgraded prognostic markers. CDH17, a liver-intestine cadherin, is an adhesion molecule implicated in tumor progression. This retrospective study assessed the immunohistochemical expression of CDH17 in 84 CRC cases with lymphovascular invasion (LVI), analyzing its correlation with clinicopathological features and survival outcomes. CDH17 expression was evaluated in the tumor core, invasive front, tumor emboli, and lymph node metastases. Statistical analyses showed significant associations between high CDH17 expression and favorable histological types, as well as low-grade differentiation. However, high CDH17 levels in tumor emboli correlated with advanced T stage and poorer overall survival. Multivariable Cox regression confirmed CDH17 expression in tumor emboli as an independent prognostic factor, indicating an approximately twofold risk of death. These findings suggest that CDH17 may have a dual role—maintaining adhesion in low-grade tumors while facilitating tumor emboli-related dissemination. CDH17 expression, particularly in the tumor emboli, could serve as a valuable prognostic biomarker in CRC with LVI.

## 1. Introduction

Malignant tumors represent a major public health concern because they are responsible for one in six of total deaths and one in four deaths from non-communicable diseases globally [1,2]. Among all malignancies, gastrointestinal tract carcinomas account for 26% of the newly discovered cases and are liable for 35% of cancer-related deaths [3]. The colorectal cancer (CRC) represents the most frequent neoplasm developed in the digestive system [2]. Therefore, ongoing research in the field of gastrointestinal tract oncology focuses on discovering, exploring, and better understanding the key points in carcinogenesis, yielding new insights to better predict, diagnose, and treat.

CRC cases diagnosed in an advanced stage display a much faster proliferation rate, an increased predisposition for invasion and metastasis, and a variability in treatment response, putting patients at high risk of recurrence [4,5,6,7].

Cadherins are adhesion molecules found in the adherens junctions, together with immunoglobulins, integrins, and selectins [8]. Among them, liver-intestine cadherin (CDH17) is regarded as a unique member, with only 20–30% similarity to classical cadherins, and included in the seven-pass transmembrane domain cadherins group [9,10]. CDH17 has been reported to be present in a wide variety of adenocarcinomas, including those of the digestive system [11,12,13,14,15,16,17,18].

Several experimental studies have shown that low levels of CDH17 in CRC are linked to invasion and metastasis [19,20,21]. However, it is yet unknown by what exact mechanism CDH17 participates in the metastasis and carcinogenesis processes. One theory states that decreased levels of CDH17 immunohistochemical expression stimulate tumor cell invasion by increasing tumor cell adherence and possible migration (by changing galectin-3 immunohistochemical expression), as well as by degrading the extracellular matrix (ECM) (through MMP-2 and MMP-9 activation) [20,22].

The study aims to examine the relationship between the CDH17 immunohistochemical profile and clinicopathological features, as well as survival outcomes, to highlight its potential prognostic role in CRC cases with lymphovascular invasion (LVI).

## 2. Results

### 2.1. CDH17 Immunohistochemical Expression and Clinicopathological Characteristics

**Figure 1:**
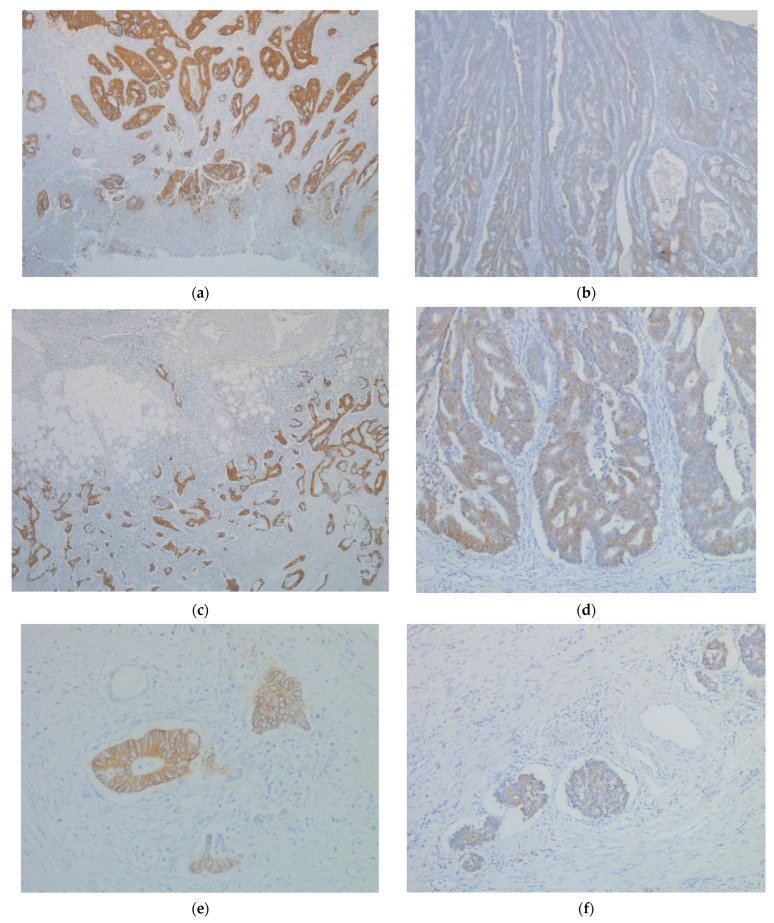

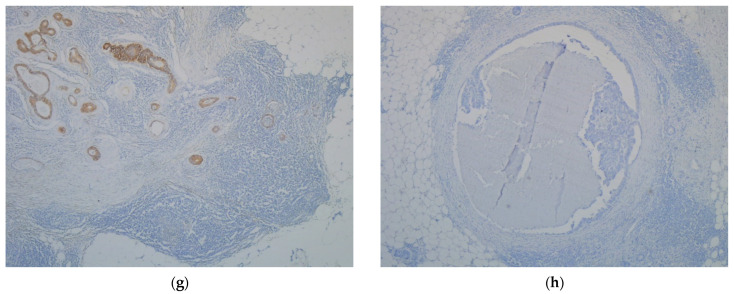
High immunohistochemical expression levels of CDH17 (IHC, anti-CDH17 monoclonal antibody) in: (**a**) tumor core (IHC, ×4); (**c**) tumor invasive front (IHC, ×4); (**e**) tumor emboli (IHC, ×20); (**g**) lymph node metastasis (IHC, ×10) versus Low immunohistochemical expression levels of CDH17 (IHC, anti-CDH17 monoclonal antibody) in: (**b**) tumor core (IHC, ×10); (**d**) tumor invasive front (IHC, ×10); (**f**) tumor emboli (IHC, ×10); (**h**) lymph node metastasis (IHC, ×4).

The immunohistochemical expression of CDH17 varied greatly in the CRC cases. Semi-quantitative score values, divided by cut-offs in low and high immunohistochemical expression subsets, confirmed the qualitative immunostaining differences observed in tumor core, tumor invasion front, tumor emboli, and in lymph node metastases (Figure 1).

Statistical analysis showed several correlations between CDH17 immunohistochemical expression (in tumor core/tumor invasive front/tumor emboli/lymph node metastases) and clinicopathological features (Table 1 and Table 2).

A significant statistical association was found between CDH17 immunohistochemical expression in tumor core/tumor emboli/lymph node metastasis and histological type (*p* = 0.001/*p* = 0.023/*p* = 0.022) (Table 1 and Table 2), with high CDH17 levels being more associated with adenocarcinoma NOS cases. Furthermore, high CDH17 levels in tumor core/tumor emboli were significantly associated with grading (*p* = 0.004/*p* = 0.040) (Table 1), especially with a low-grade of differentiation.

CDH17 immunohistochemical expression in the tumor core was statistically significantly associated with EPNI (*p* = 0.041) and Bd (*p* = 0.037) (Table 1). Specifically, low levels of CDH17 immunohistochemical expression in the tumor core were significantly more correlated with the presence of EPNI, whereas high levels of CDH17 immunohistochemical expression were significantly more correlated with a low score of Bd (Bd1). However, CDH17 immunohistochemical expression in the tumor core was not associated with the other clinicopathological features (*p* > 0.05) (Table 1).

CDH17 immunohistochemical expression in the tumor invasive front was significantly associated only with PDCs (*p* = 0.018) (Table 1), which suggests that low CDH17 levels are considerably more correlated with a score of Pdc2. The remaining evaluated clinicopathological features (gender, T stage, N status, grading, LVI, EMVI, IMVI, EPNI, IPNI, tumor growth pattern, and Bd) showed no significant statistical association (*p* > 0.05) (Table 1).

Additionally, a strong statistical association was found between CDH17 immunohistochemical expression in tumor emboli and T stage (*p* = 0.046) (Table 1), suggesting that high CDH17 levels in tumor emboli are more associated with T stage 3. In contrast, CDH17 immunohistochemical expression in tumor emboli was not correlated with the remaining clinicopathological features (*p* > 0.05) (Table 1).

### 2.2. CDH17 Immunohistochemical Expression and Survival

**Figure 2:**
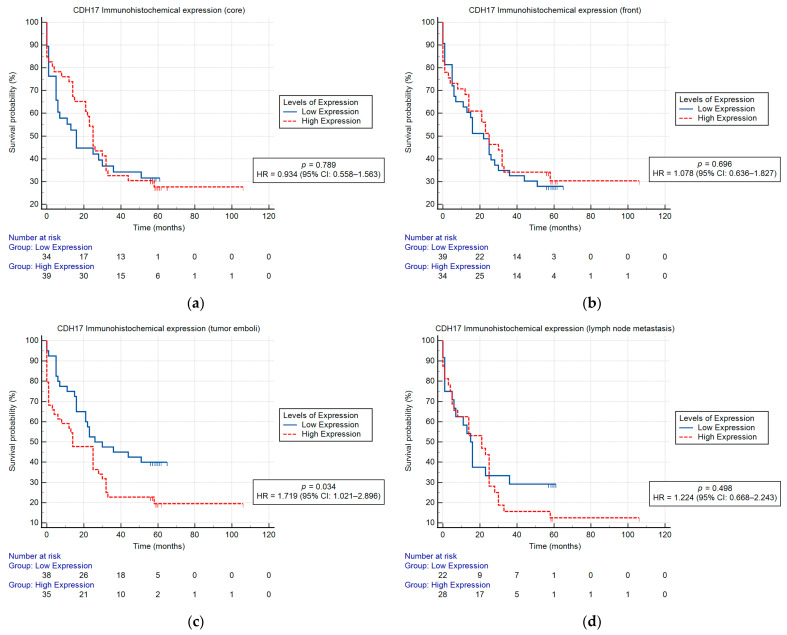
Kaplan–Meier curves showing OS for CRC patients according to CDH17 immunohistochemical expression in tumor core (**a**), tumor invasive front (**b**), tumor emboli (**c**), and lymph node metastasis (**d**); a significant correlation was found between CDH17 immunohistochemical expression in tumor emboli and OS (***p* = 0.034**).

The Kaplan–Meier univariate analysis showed a statistically significant correlation between CDH17 immunohistochemical expression and OS in tumor emboli (*p* = 0.034), high CDH17 levels signifying a poor prognosis. The Kaplan–Meier univariate survival analysis indicated that the CDH17 immunohistochemical expression in the tumor core, the tumor invasive front, and lymph node metastasis had no effect on OS (Figure 2). Moreover, the univariable Cox proportional hazard analysis showed a significant correlation between CDH17 immunohistochemical expression and OS in N status (*p* = 0.007), LVI (*p* = 0.026), EMVI (*p* = 0.014), EPNI (*p* = 0.012), tumor emboli (*p* = 0.042), and prognostic stage group (*p* = 0.007) (Table 3).

Given that the study group had 59 events (deaths), a multivariable Cox proportional hazards model might incorporate a maximum of 6 potential predictors. The selected predictors used in the model were CDH17 immunohistochemical expression in tumor emboli, histological type, LVI, EMVI, EPNI, and prognosis stage group. The Omnibus Tests of Model Coefficients for the Cox multivariable analysis demonstrated statistical significance (*p* = 0.004) for the developed model. The results indicated that the high levels of CDH17 immunohistochemical expression in tumor emboli (*p* = 0.028), with a hazard rate (HR) of 2.003 (95% CI: 1.077–3.723), may function as an independent prognostic factor (Table 3). Our data showed that the hazard rate (HR) for mortality was significantly elevated in patients exhibiting high levels of CDH17 immunohistochemical expression compared to those with low levels of CDH17 immunohistochemical expression. Our data showed that patients with high levels of CDH17 immunohistochemical expression in tumor emboli have an approximately twofold risk of death compared to those with low expression, adjusted for other covariates.

## 3. Discussion

To our knowledge, there are a limited number of studies examining the relationship between CDH17 immunohistochemical expression and a comprehensive range of clinicopathological features [23,24], as well as survival outcomes [24] in CRC. The review of the literature shows a focus on CDH17 from other perspectives, analyzing the diagnostic value and prognostic significance of serum CDH17 (together with serum miR-378e) [25], the importance of single-nucleotide polymorphisms in the CDH17 gene [26,27], and the effectiveness of identifying CDH17 in circulating tumor cells compared to tissue immunoexpression for the non-invasive detection of CRC [28].

Thus, we underline that our study represents the first analysis of CDH17 immunohistochemical expression across the tumor core, tumor invasive front, tumor emboli, and lymph node metastasis in relation to clinicopathological features and survival outcomes, and its potential involvement as a prognostic factor in CRC.

Our results indicated a statistically significant association between high CDH17 immunohistochemical expression in the tumor core/tumor emboli/lymph node metastasis and the adenocarcinoma NOS histological type. These findings essentially prove a greater ability to maintain cell adhesion through CDH17 in the most common histological type of CRC, namely, adenocarcinoma NOS, which has a better prognosis than other histological types. Therefore, at first glance, CDH17 may be associated with less aggressive tumor behavior. The tumor behavior analysis requires a connection between histological type and the degree of differentiation, and our results indicate a statistically significant association between CDH17 immunohistochemical expression in the tumor core/tumor emboli and grading, with CDH17 high levels characterizing low-grade (moderate and well-differentiated) CRC. This fact highlights CDH17’s role in the intrinsic cellular mechanism of tumor progression; the phenotypic characteristics responsible for a low-grade behavior depend on the functionality of intercellular junctions. Our results are in concordance with one of the few studies analyzing the CDH17 profile in CRC, which reports a link between high levels of CDH17 immunohistochemical expression and moderate and well-differentiated cases (low-grade), whereas diminished levels correlate with poorly differentiated cases (high-grade) [23]. Recent results regarding CDH17 serum levels in CRC are also in line with this finding [25]. Considering that serum CDH17 levels mirror the tissue framework of this intercellular adhesion molecule, the above-mentioned study provides clear evidence to support that well-differentiated, low-grade tumors preserve high CDH17 immunohistochemical expression, whereas those considered high-grade, due to poor cellular differentiation, show decreased one [25].

To the best of our knowledge, our study brings novelty by examining CDH17 in relationship to EPNI, Bd, and PDCs—additional prognostic factors included in the histopathological evaluation and reporting according to the latest edition of the Digestive System Tumours: WHO Classification of Tumours [29]. It is worth noticing that the literature does not include reports on the CDH17 profile correlated with these supplementary prognostic factors. Hence, we noticed new associations among CDH17 immunohistochemical expression in the tumor core and EPNI and Bd, respectively. These results can explain the extent of tumor migration and invasion processes from the tumor core and, consequently, EPNI through the loss of intercellular adhesion, indicated by low CDH17 levels, or the limitation of these processes by maintaining high CDH17 levels, and, subsequently, a low Bd score (Bd1).

For the first time, we demonstrated that CDH17 immunohistochemical expression at the invasion front had a statistically significant association with PDCs, low CDH17 levels being correlated with a score of Pdc2. This fact provides further evidence of the prognostic significance of this adhesion molecule, whose loss may determine the appearance of PDCs.

Nonetheless, our study analyzes the CDH17 immunohistochemical expression in tumor emboli, showing a statistically significant association with T stage. Surprisingly, high CDH17 immunohistochemical expression in tumor emboli was correlated with T3 stage. A possible explanation for keeping CDH17 levels in tumor emboli, even when tumor staging is T3, may be related to the early stages of their formation—detachment of tumor cells is possible through the loss of other adhesion molecules, while CDH17 remains stable.

Aside from the associations previously noted, we did not obtain statistically significant results for CDH17 immunohistochemical expression in tumor core/invasive front/tumor emboli/lymph node metastasis and other clinicopathological characteristics, such as gender, N status, LVI, IMVI, EMVI, IPNI, prognostic stage group, and tumor growth pattern.

From this perspective, our data are inconsistent with other studies analyzing CDH17 immunohistochemical expression, which show an association between low CDH17 and LVI, lymph node metastasis, and advanced stage [23], or between high CDH17 and distant metastases and advanced stage [28,30]. These inconsistencies can be supplemented by an investigation focusing on serum CDH17 levels, which report correlations with lymph node metastasis and clinical stage [28]. In our opinion, these contradictory results are a consequence of the great variability in methodological approaches [23,28,30], involving different structures of the study groups—such as the number of patients and/or analyzed parameters—and in the different systems used for evaluating immunoreactivity, including the use of digital image analysis [30].

In terms of methodology, it is worth detailing a recent study based on an automated algorithm developed using digital image analysis for quantifying CDH17 in 150 retrospective CRC cases [30]. The authors proposed a digital scoring method that results from a semi-quantitative assessment of membrane staining, incorporating both the staining intensity (i) and percentage of stained cells at each intensity level (Pi), with a calculated range from 0 to 50. The automated algorithm was built by analyzing more than 10,000 cells for each case, and the obtained digital Membrane (M) Score was validated through a strong positive correlation with a manual score established by a pathologist. The benefit of an automated determination is undeniable; however, it should be considered that the efficiency of the automated evaluation has been validated by comparison with a manual score, such as the one used by us. The authors found that high CDH17 expression in tumor tissue was associated with advanced tumor staging and distant metastasis. In contrast, our study analyzed CDH17 immunoexpression in multiple histological regions—tumor core, invasive front, lymphovascular emboli, and lymph node metastases—in 84 CRC cases selected for the presence of lymphovascular invasion. These design differences likely account for the result’s divergences, with our study confirming a significant association between high CDH17 expression and histological type, grade, and Bd in the tumor core, as well as advanced stage in tumor emboli.

The framework for investigating the CDH17 profile in CRC and its relationship with clinicopathological characteristics was expanded by including patient survival as a major endpoint for assessing the prognostic influence of this biomarker on tumor behavior. As mentioned above, the literature includes only three studies in which the analysis of CDH17 tissue or serum levels includes correlations with survival parameters [24,25,30]. Published data show that the survival of patients with high CDH17 serum levels is statistically significantly higher compared to those with low CDH17 serum levels [25]. On the other hand, the other studies based on the evaluation of CDH17 tissue immunohistochemical expression highlight that high CDH17 is associated with poor OS and recurrence-free survival and is confirmed as an independent prognostic predictor for both survival outcomes [24,30]. We reiterate that our study analyzed CDH17 expression in four different locations: the tumor core, the tumor invasive front, the tumor emboli, and the lymph node metastasis, thus providing comprehensive insight into the dynamics of this intercellular adhesion molecule. Through the Kaplan–Meier univariate analysis, we found a statistically significant correlation between CDH17 immunohistochemical expression in tumor emboli and OS, with high CHD17 being correlated with a poor prognosis. Moreover, the multivariable Cox proportional hazard model demonstrated that CDH17 immunohistochemical expression in tumor emboli may act as an independent prognostic factor. Nonetheless, we demonstrate that patients with high levels of CDH17 immunohistochemical expression in tumor emboli have an approximately twofold risk of death compared to those with low expression, adjusted for other covariates. To the best of our knowledge, our study is the first report to sustain high CDH17 levels in tumor emboli as an independent prognostic biomarker in CRC. Additionally, our results agree with those reported in the literature, considering CDH17 immunohistochemical expression throughout the entire tumor mass [24,30]. Therefore, we reckon that the routine, consistent assessment of CDH17 immunohistochemical expression in CRC may become an additional criterion in prognostic evaluation and, at the same time, in the development of therapeutic possibilities, given the current research targeting CDH17 as a potential treatment [31,32,33,34,35].

## 4. Materials and Methods

### 4.1. Study Group

A total of 84 patients histologically diagnosed with both CRC and LVI, between June 2013 and December 2018, were included in the retrospective analysis. Each patient gave their written informed consent, and the Ethics Committees of the University of Medicine and Pharmacy “Grigore T. Popa” Iasi, Romania, and the “Sf. Spiridon” Emergency County Hospital Iasi, Romania, approved this study (No. 43/10.02.2021).

Inclusion criteria were (1) a confirmed diagnosis of CRC and (2) the presence of lymphovascular invasion, as identified by hematoxylin-eosin staining and confirmed by histopathological assessment. Cases without lymphovascular invasion or incomplete clinical data were excluded.

### 4.2. Pathological Reassessment

All cases were reassessed according to the updating of histopathological classification, grading, and staging landmarks [29,36]. We registered the following clinicopathological features: histological type, tumor (T) stage, node (N) status, grading, LVI, extramural and intramural vascular invasion (EMVI/IMVI), extramural and intramural perineural invasion (EPNI/IPNI), tumor growth pattern, tumor budding (Bd), and poorly differentiated clusters (PDCs) (Table 4). Supplementary, we included data regarding patient overall survival (OS), defined as the time between a pathological diagnosis and death (Table 4). Patients were followed up between June 2013 and March 2023, with a median OS rate of 24 months. At the end of the follow-up period, 25 (29.8%) patients were still alive. All patients’ deaths were related to cancer.

### 4.3. Immunohistochemistry

The anti-CDH17 monoclonal antibody (rabbit anti-human, 1:100, clone 29, MA5-29136, Invitrogen, Waltham, MA, USA) was used for the immunohistochemical analysis following a 5-min protein digestion with proteinase K. UltraVision LP Detection System (ThermoFisher Scientific, Fremont, CA, USA) and 3,3′-Diaminobenzidine chromogen (DAB) (ThermoFisher Scientific, Fremont, CA, USA) were utilized for the immunoreaction detection. In every run, controls were included. Positive control was represented by the staining of the intestinal glands of the appendix from patients with non-tumor pathology. Negative control was obtained by replacing the primary antibody with wash buffer.

CDH17 immunohistochemical membranous staining of tumor cells was considered positive. The percentage of positive tumor cells and the intensity of immunoreaction was assessed using a four-tiered scoring system as follows: 0 = 0% positive tumor cells, 1 = <10% positive tumor cells, 2 = 11–50% positive tumor cells, 3 = 51–80% positive tumor cells, and 4 = 81–100% positive tumor cells; 0 = negative reaction, 1 = weak intensity, 2 = moderate intensity, and 3 = strong intensity [37]. The proportion and intensity scores were multiplied to determine the total immunoreactivity score (IRS). Consequently, the IRS varied between 0 and 12 [37]. The immunoreaction was assessed in the tumor core and invasive front, as well as in tumor emboli and lymph node metastases.

Initially, receiver operating characteristic (ROC) curve analysis was used to decide the optimal cut-off value for low- and high-levels of CDH17 expression, but due to the fact that the area under the receiver operating characteristic curve (AUC) had a poor discrimination and the *p* value was not statistically significant, a cut-off value could not be determined. Thus, another approach was made by taking into consideration the analysis of IRS distribution by density plots, using a Kernel density estimate plot, and choosing curve points suitable for displaying two subsets of variables after asymmetrical distributions. Since none of the evaluated sites—tumor core, tumor invasive front, tumor emboli, and lymph node metastasis—had a distinct discrimination point, the median CDH17 immunohistochemical expression value was deemed the ideal cut-off value. Therefore, for CDH17 immunohistochemical expression in tumor core, a cut-off value of 7 was found (IRS < 7—low immunohistochemical expression/IRS ≥ 7—high immunohistochemical expression) and for CDH17 immunohistochemical expression in tumor invasive front, tumor emboli and lymph node metastases, a cut-off value of 6 was established (IRS < 6—low immunohistochemical expression/IRS ≥ 6—high immunohistochemical expression).

The evaluation was conducted by two separate pathologists in a blinded manner. In cases with conflicting evaluation outcomes, a panel assessment was conducted in order to reach a consensus.

### 4.4. Statistical Analysis

The statistical analysis was carried out with Microsoft Office Excel, IBM Statistical Package for the Social Sciences (SPSS) version 26 (IBM Corporation, Armonk, New York, NY, USA), and MedCalc for Windows, version 20.118 (MedCalc Software, Ostend, Belgium). In order to determine whether there is an association or a correlation between levels of CDH17 immunohistochemical expression and clinicopathological features, the Chi-Square Test of Independence and Fisher’s exact test were used. The Kaplan–Meier procedure (log-rank test) was used to estimate OS for univariate survival. To determine the OS hazard ratios and confidence intervals, both univariately and multivariable, the Cox proportional hazard model was used. Based on a statistically significant *p*-value (<0.05) and clinical importance, the clinicopathological features for the multivariable analysis were chosen.

## 5. Conclusions

Our research provides novel insights into CDH17 analysis in CRC through a complex assessment targeting the tumor core, invasive front, tumor emboli, and lymph node metastasis, as well as their relationship with a large panel of clinicopathological and survival characteristics. High levels of CDH17 immunohistochemical expression in tumor emboli were confirmed as an independent prognostic biomarker, being correlated with a poor outcome and doubling the risk of death.

## Figures and Tables

**Table 1:** Univariate analysis of clinicopathological parameters by CDH17 expression level (Low vs. High) in the tumor core, invasion front, and tumor emboli.

Clinicopathological Parameters		*n* 84	CDH17 (Core) Expression		CDH17 (Front) Expression		CDH17 (Emboli) Expression	
			Low	High	Low	High	Low	High
Gender	male	43	16 (19%)	27 (32.1%)	19 (22.6%)	24 (28.6%)	20 (23.8%)	23 (27.4%)
	female	41	22 (26.2%)	19 (22.6%)	24 (28.6%)	17 (20.2%)	20 (23.8%)	21 (25%)
			*p* = 0.130		*p* = 0.188		*p* = 0.835	
	ADK NOS	64	23 (27.4%)	41 (48.8%)	29 (34.5%)	35 (41.7%)	25 (29.8%)	39 (46.4%)
	ADK with mucinous component	13	11 (13.1%)	2 (2.4%)	9 (10.7%)	4 (4.8%)	10 (11.9%)	3 (3.6%)
Histological type	ADK with signet ring cell component	2	2 (2.4%)	0 (0%)	2 (2.4%)	0 (0%)	1 (1.2%)	1 (1.2%)
	medullary ADK	1	1 (1.2%)	0 (0%)	1 (1.2%)	0 (0%)	1 (1.2%)	0 (0%)
	mucinous ADK	4	1 (1.2%)	3 (3.6%)	2 (2.4%)	2 (2.4%)	3 (3.6%)	1 (1.2%)
			***p*** **= 0.001**		*p* = 0.227		***p*** **= 0.023**	
Grade	high-grade	12	10 (11.9%)	2 (2.4%)	9 (10.7%)	3 (3.6%)	9 (10.7%)	3 (3.6%)
	low-grade	72	28 (33.3%)	44 (52.4%)	34 (40.5%)	38 (45.2%)	31 (36.9%)	41 (48.8%)
			***p*** **= 0.004**		*p* = 0.075		***p*** **= 0.040**	
	1	1	0 (0%)	1 (1.2%)	0 (0%)	1 (1.2%)	0 (0%)	1 (1.2%)
	2	2	1 (1.2%)	1 (1.2%)	2 (2.4%)	0 (0%)	2 (2.4%)	0 (0%)
T stage								
	3	43	16 (19%)	27 (32.1%)	18 (21.4)	25 (29.8%)	16 (19%)	27 (32.1%)
	4	38	21 (25%)	17 (20.2%)	23 (27.4%)	15 (17.9%)	22 (26.2%)	16 (19%)
			*p* = 0.240		*p* = 0.083		***p*** **= 0.046**	
	negative	28	10 (11.9%)	18 (21.4%)	14 (16.7%)	14 (16.7%)	16 (19%)	12 (14.3%)
N status								
	positive	56	28 (33.3%)	28 (33.3%)	29 (34.5%)	27 (32.1%)	24 (28.6%)	32 (38.1%)
			*p* = 0.215		*p* = 1.000		*p* = 0.217	
	LVI2	27	13 (15.5%)	14 (16.7%)	12 (14.3%)	15 (17.9%)	17 (20.2%)	10 (11.9%)
LVI								
	LVI4	57	25 (29.8%)	32 (38.1%)	31 (36.9%)	26 (31%)	23 (27.4)	34 (40.5%)
			*p* = 0.712		*p* = 0.395		*p* = 0.053	
IMVI	absent	61	28 (33.3%)	33 (39.3%)	28 (33.3%)	33 (39.3%)	31 (36.9%)	30 (35.7%)
	present	23	10 (11.9%)	13 (15.5%)	15 (17.9%)	8 (9.5%)	9 (10.7%)	14 (16.7%)
			*p* = 0.842		*p* = 0.114		*p* = 0.339	
EMVI	absent	37	16 (19%)	21 (25%)	20 (23.8%)	17 (20.2%)	22 (26.2%)	15 (17.9%)
	present	47	22 (26.2%)	25 (29.8%)	23 (27.4%)	24 (28.6%)	18 (21.4%)	29 (34.5%)
			*p* = 0.744		*p* = 0.641		*p* = 0.054	
IPNI	absent	52	22 (26.2%)	30 (35.7%)	23 (27.4%)	29 (34.5%)	25 (29.8%)	27 (32.1%)
	present	32	16 (19%)	16 (19%)	20 (23.8%)	12 (14.3%)	15 (17.9%)	17 (20.2%)
			*p* = 0.492		*p* = 0.104		*p* = 0.915	
EPNI	absent	39	13 (15.5%)	26 (31%)	21 (25%)	18 (21.4%)	19 (22.6%)	20 (23.8%)
	present	45	25 (29.8%)	20 (23.8%)	22 (26.2%)	23 (27.4%)	21 (25%)	24 (28.6%)
			***p*** **= 0.041**		*p* = 0.650		*p* = 0.851	
Prognostic stage group	I-II	28	10 (11.9%)	18 (21.4%)	14 (16.7%)	14 (16.7%)	16 (19%)	12 (14.3%)
	III-IV	56	28 (33.3%)	28 (33.3%)	29 (34.5%)	27 (32.1%)	24 (28.6%)	32 (38.1%)
			*p* = 0.215		*p* = 0.877		*p* = 0.217	
Tumor growth pattern	infiltrative	71	31 (36.9%)	40 (47.6%)	35 (41.7%)	36 (42.9%)	35 (41.7%)	36 (42.9%)
	pushing borders	13	7 (8.3%)	6 (7.1%)	8 (9.5%)	5 (6%)	35 (41.7%)	36 (42.9%)
			*p* = 0.498		*p* = 0.417		*p* = 0.472	
Bd	Bd1	48	16 (19%)	32 (38.1%)	20 (23.8%)	28 (33.3%)	21 (25%)	27 (32.1%)
	Bd2	19	11 (13.1%)	8 (9.5%)	12 (14.3%)	7 (8.3%)	12 (14.3%)	7 (8.3%)
	Bd3	17	11 (13.1%)	6 (7.1%)	11 (13.1%)	6 (7.1%)	7 (8.3%)	10 (11.9%)
			***p*** **= 0.037**		*p* = 0.130		*p* = 0.300	
PDCs	Pdc1	54	21 (25%)	33 (39.3%)	24 (28.6%)	30 (35.7%)	24 (28.6%)	30 (35.7%)
	Pdc2	25	14 (16.7%)	11 (13.1%)	18 (21.4%)	7 (8.3%)	13 (15.5%)	12 (14.3%)
	Pdc3	5	3 (3.6%)	2 (2.4%)	1 (1.2%)	4 (4.8%)	3 (3.6%)	2 (2.4%)
			*p* = 0.309		***p*** **= 0.018**		*p* = 0.710	

*n*, total number of cases; *p*-value < 0.05; ADK, adenocarcinoma; LVI, lymphovascular invasion; IMVI, intramural vascular invasion; EMVI, extramural vascular invasion; IPNI, intramural perineural invasion; EPNI, extramural perineural invasion; Bd, tumor budding; PDCs, poorly differentiated clusters. Comparisons were performed using Chi-square or Fisher’s exact test between CDH17 low and high expression groups. *p*-values are shown beneath each variable group.

**Table 2:** Univariate analysis of clinicopathological parameters by CDH17 expression level (Low vs. High) in lymph node metastasis.

Clinicopathological Parameters		*n* 56	CDH17 Expression (Lymph Node Metastasis)	
			Low	High
Gender	male	27	9 (16.1%)	18 (32.1%)
	female	29	15 (26.8%)	14 (25%)
			*p* = 0.165	
Histological Type	ADK NOS	40	15 (26.8%)	25 (44.6%)
	ADK with mucinous component	10	7 (12.5%)	3 (5.4%)
	ADK with signet ring cell component	1	1 (1.8%)	0 (0%)
	medullary ADK	1	1 (1.8%)	0 (0%)
	mucinous ADK	4	0 (0%)	4 (7.1%)
			***p*** **= 0.022**	
Grade	high-grade	8	4 (7.1%)	4 (7.1%)
	low-grade	48	20 (35.7%)	28 (50%)
			*p* = 0.713	
T stage	1	0	0 (0%)	0 (0%)
	2	0	0 (0%)	0 (0%)
	3	25	8 (14.3%)	17 (30.4%)
	4	31	16 (28.6%)	15 (26.8%)
			*p* = 0.140	
LVI	LVI2	12	6 (10.7%)	6 (10.7%)
	LVI4	44	18 (31.1%)	26 (46.4%)
			*p* = 0.573	
IMVI	absent	38	17 (30.4%)	21 (37.5%)
	present	18	7 (12.5%)	11 (19.6%)
			*p* = 0.680	
EMVI	absent	18	9 (16.1%)	9 (16.1%)
	present	38	15 (26.8%)	23 (41.1%)
			*p* = 0.457	
IPNI	absent	32	13 (23.2%)	19 (33.9%)
	present	24	11 (19.6%)	13 (23.2%)
			*p* = 0.697	
EPNI	absent	18	6 (10.7%)	12 (21.4%)
	present	38	18 (32.1%)	20 (35.7%)
			*p* = 0.322	
Prognostic stage group	I-II	0	0 (0%)	0 (0%)
	III-IV	56	24 (42.9%)	32 (57.1%)
			a	
Tumor growth pattern	infiltrative	48	20 (35.7%)	28 (50%)
	pushing borders	8	4 (7.1%)	4 (7.1%)
			*p* = 0.713	
Bd	Bd1	25	9 (16.1%)	16 (28.6%)
	Bd2	15	10 (17.9%)	5 (8.9%)
	Bd3	16	5 (8.9%)	11 (19.6%)
			*p* = 0.089	
PDCs	Pdc1	31	12 (21.4%)	19 (33.9%)
	Pdc2	22	11 (19.6%)	11 (19.6%)
	Pdc3	3	1 (1.8%)	2 (3.6%)
			*p* = 0.747	

*n*, total number of cases; *p*-value < 0.05; ADK, adenocarcinoma; LVI, lymphovascular invasion; IMVI, intramural vascular invasion; EMVI, extramural vascular invasion; IPNI, intramural perineural invasion; EPNI, extramural perineural invasion; Bd, tumor budding; PDCs, poorly differentiated clusters; a, no statistics are computed because Prognostic stage group is a constant; Comparisons were performed using Chi-square or Fisher’s exact test between CDH17 low and high expression groups. *p*-values are shown beneath each variable group.

**Table 3:** Univariate and multivariable Cox regression analysis of clinicopathological features for OS in patients with CRC.

Variables	Univariate Analysis	*p*-Value	Multivariable Analysis	*p*-Value
	HR/CI (95%)		HR/CI (95%)	
Gender (male/female)	0.963 (0.577–1.607)	0.896		
Histological type (ADK NOS/Other subtypes)	1.502 (0.832–2.713)	0.177		
Grade (high-grade/low-grade)	0.757 (0.372–1.543)	0.444		
T stage (1/2/3/4)	1.157 (0.765–1.751)	0.490		
N status (negative/positive)	2.300 (1.254–4.220)	**0.007**		
LVI (LVI2/LVI4)	1.952 (1.084–3.518)	**0.026**		
IMVI (absent/present)	1.442 (0.827–2.514)	0.197		
EMVI (absent/present)	1.954 (1.148–3.324)	**0.014**		
IPNI (absent/present)	0.897 (0.528–1.521)	0.686		
EPNI (absent/present)	1.959 (1.160–3.307)	**0.012**		
Prognostic stage group (I-II/III-IV)	2.300 (1.254–4.220)	**0.007**		
Tumor growth pattern (infiltrative/pushing borders)	1.346 (0.698–2.595)	0.376		
Bd1	1.000 (Reference)			
Bd2	1.286 (0.686–2.412)	0.433		
Bd3	1.299 (0.680–2.481)	0.428		
Pdc1	1.000 (Reference)			
Pdc2	1.414 (0.814–2.456)	0.219		
Pdc3	1.112 (0.342–3.615)	0.859		
CDH17 expression (core) (low/high)	0.934 (0.558–1.563)	0.795		
CDH17 expression (front) (low/high)	1.078 (0.636–1.827)	0.782		
CDH17 expression (emboli) (low/high)	1.719 (1.021–2.896)	**0.042**	2.003 (1.077–3.723)	**0.028**

*p*-value < 0.05; HR, hazard rate; CI, confidence interval; T stage was considered as a numeric variable. ADK, adenocarcinoma; LVI, lymphovascular invasion; IMVI, intramural vascular invasion; EMVI, extramural vascular invasion; IPNI, intramural perineural invasion; EPNI, extramural perineural invasion; Bd, tumor budding; PDCs, poorly differentiated clusters.

**Table 4:** Summary of the main clinicopathological characteristics and survival outcome.

Clinicopathological Parameters		*n* (%)
Gender	male	43 (51.2%)
	female	41 (48.8%)
Histological type	ADK NOS	64 (76.2%)
	ADK with mucinous component	13 (15.5%)
	ADK with signet ring cell component	2 (2.4%)
	medullary ADK	1 (1.2%)
	mucinous ADK	4 (4.8%)
Grade	high-grade	12 (14.3%)
	low-grade	72 (85.7%)
T stage	1	1 (1.2%)
	2	2 (2.4%)
	3	43 (51.2%)
	4	38 (45.2%)
N status	negative	28 (33.3%)
	positive	56 (66.7%)
LVI	LVI2	27 (32.1%)
	LVI4	57 (67.9%)
IMVI	absent	61 (72.6%)
	present	23 (27.4%)
EMVI	absent	37 (44%)
	present	47 (56%)
IPNI	absent	52 (61.9%)
	present	32 (38.1%)
EPNI	absent	39 (46.4%)
	present	45 (53.6%)
Prognostic stage group	I-II	28 (33.3%)
	III-IV	56 (66.7%)
Tumor growth pattern	infiltrative	71 (84.5%)
	pushing borders	13 (15.5%)
Bd	Bd1	48 (57.1%)
	Bd2	19 (22.6%)
	Bd3	17 (20.2%)
PDCs	Pdc1	54 (64.3%)
	Pdc2	25 (29.8%)
	Pdc3	5 (6%)
Overall survival	alive	25 (29.8%)
	dead	59 (70.2%)

*n*, total number of cases; ADK, adenocarcinoma; LVI, lymphovascular invasion; IMVI, intramural vascular invasion; EMVI, extramural vascular invasion; IPNI, intramural perineural invasion; EPNI, extramural perineural invasion; Bd, tumor budding; PDCs, poorly differentiated clusters.

## Data Availability

Data supporting this study are not publicly available due to ethical restrictions. Please contact the corresponding author for further information.
